# Prediction of Ubiquitination Sites by Using the Composition of *k*-Spaced Amino Acid Pairs

**DOI:** 10.1371/journal.pone.0022930

**Published:** 2011-07-29

**Authors:** Zhen Chen, Yong-Zi Chen, Xiao-Feng Wang, Chuan Wang, Ren-Xiang Yan, Ziding Zhang

**Affiliations:** 1 State Key Laboratory of Agrobiotechnology, College of Biological Sciences, China Agricultural University, Beijing, China; 2 Bioinformatics Center, College of Biological Sciences, China Agricultural University, Beijing, China; 3 Tianjin Cancer Institute, Tianjin Medical University Cancer Institute and Hospital, Tianjin, China; King's College, London, United Kingdom

## Abstract

As one of the most important reversible protein post-translation modifications, ubiquitination has been reported to be involved in lots of biological processes and closely implicated with various diseases. To fully decipher the molecular mechanisms of ubiquitination-related biological processes, an initial but crucial step is the recognition of ubiquitylated substrates and the corresponding ubiquitination sites. Here, a new bioinformatics tool named CKSAAP_UbSite was developed to predict ubiquitination sites from protein sequences. With the assistance of Support Vector Machine (SVM), the highlight of CKSAAP_UbSite is to employ the composition of *k*-spaced amino acid pairs surrounding a query site (i.e. any lysine in a query sequence) as input. When trained and tested in the dataset of yeast ubiquitination sites (Radivojac et al, *Proteins*, 2010, 78: 365–380), a 100-fold cross-validation on a 1∶1 ratio of positive and negative samples revealed that the accuracy and MCC of CKSAAP_UbSite reached 73.40% and 0.4694, respectively. The proposed CKSAAP_UbSite has also been intensively benchmarked to exhibit better performance than some existing predictors, suggesting that it can be served as a useful tool to the community. Currently, CKSAAP_UbSite is freely accessible at http://protein.cau.edu.cn/cksaap_ubsite/. Moreover, we also found that the sequence patterns around ubiquitination sites are not conserved across different species. To ensure a reasonable prediction performance, the application of the current CKSAAP_UbSite should be limited to the proteome of yeast.

## Introduction

As one of the most important reversible protein post-translational modifications (PTMs), ubiquitination occurs when ubiquitin (Ub) is covalently attached to lysine (K) residues of targeting proteins (i.e. ubiquitylated substrates). Three enzymes are implicated in the process of ubiquitination, including Ub-activating (E1), Ub-conjugating (E2) and Ub-ligating (E3) enzymes, and the types of ubiquitination are diverse (e.g. the targeting proteins can be linked with a single Ub or poly-Ub chains) [1]–[4]. Ubiquitination has been reported to be involved in regulating a variety of basic cellular processes, including the degradation of protein [5], [6], gene transcription, DNA repair and replication, intracellular trafficking and virus budding [1]. Meanwhile, increasing evidences have also demonstrated that the change of the ubiquitination system is closely related with cellular transformation, immune response and inflammatory response [7]. Of the aforementioned functional roles, the regulatory function of the Ub-proteasome system is certainly of utmost significance for cellular homeostasis. About 80% of the cellular proteins are degraded by the Ub-proteasome system [8].

To decipher the mechanism of Ub-proteasome system or other regulatory roles of ubiquitination at the molecular level, an initial but crucial step is to identify ubiquitylated substrates and the corresponding ubiquitination sites [1]. Researchers have employed several experimental methods to purify ubiquitylated proteins such as the use of affinity-tagged Ub, Ub antibodies and Ub-binding proteins, and high-throughput mass-spectrometry (MS) technique [9], [10]. So far, hundreds of ubiquitylated proteins and the corresponding ubiquitination sites have been experimentally determined [10], [11], which have been further compiled into some user-friendly databases such as UbiProt (http://ubiprot.org.ru/) [12], SCUD (http://scud.kaist.ac.kr) [13] and SysPTM (http://www.sysbio.ac.cn/SysPTM) [14]. Although the specific molecular mechanism of Ub conjugation reaction to ubiquitylated substrates remains elusive [2], the accumulated data have strengthened our fundamental understanding of the sequence/structural characteristics around ubiquitination sites. Catic and co-workers (2004) systematically analyzed 135 ubiquitination sites in 95 yeast proteins [15]. From the structural context, they found that ubiquitination sites preferred to be exposed at the molecular surface and reside in loop regions [15]. Regarding the sequence context, they also discovered a sequence motif ‘KEEE’, which may be frequently employed for the attachment of Ub in yeast [15]. In 2010, Radivojac et al also analyzed the structural context of ubiquitination sites and confirmed that these sites were preferentially located in intrinsically disordered regions [2].

Considering that ubiquitination is rapid and reversible, the large-scale identification of ubiquitylated proteins and ubiquitination sites is labor-intensive and time-consuming. Parallel to the experimental identification of ubiquitination sites, there is still a serious need for bioinformatics methods to predict potential ubiquitination sites in query proteins. Similar to the development of other PTM site predictors [16]–[19], the input for an ubiquitination site predictor is generally presented by a sequence fragment of 2*n* +1 residues with the residue K in the central position (i.e. the window size is equal to 2*n*+1). An appropriate feature construction or encoding scheme of the sequence fragment is further required for the processing of a prediction algorithm. Finally, a predictor can be established by some statistical- or machine learning-based algorithms.

Up to now, several ubiquitination site prediction methods have been developed elegantly. Tung and Ho (2008) [3] developed an ubiquitination site predictor (UbiPred) using a Support Vector Machine (SVM) with 31 informative physicochemical features selected from the published amino acid indices [20]. In 2010, Radivojac et al also proposed a random forest-based predictor called UbPred, in which 586 sequence attributes were employed as the input feature vector [2]. Very recently, Cai et al developed a nearest neighbor algorithm-based ubiquitination site predictor [21]. They identified key components from 541 features and used the incremental feature selection method procedure to maximize the predictor performance [21]. It is worth mentioning that the practical applications of these established predictors have already been exploited and some prediction results have been converted into new biological findings. For instance, UbPred was employed for a proteome-wide ubiquitination site prediction in yeast [2]. Based on the prediction results, it was established that highly ubiquitylated proteins were enriched among transcription/enzyme regulators and proteins involved in cell cycle control [2].

The overall performance of the aforementioned three exciting predictors is still not fully satisfactory and there is still room to improve the predictive accuracy. In this study, we focused on developing a new ubiquitination site predictor by seeking a more informative encoding scheme. After our preliminary assessment of different encoding schemes, we found that the composition of *k*-spaced amino acid pairs (CKSAAP) is suitable for representing the sequence context surrounding the ubiquitination sites. CKSAAP reflects the short range interactions of residues within a sequence or a sequence fragment, which has been successfully employed for the prediction of protein flexible/rigid regions [22], protein crystallization [23], protein structural classes [24], membrane protein types [25]–[27], mucin-type O- glycosylation sites [16], palmitoylation sites [28], etc. With the assistance of SVM, we proposed a predictor called CKSAAP_UbSite to detect ubiquititnation sites in query proteins. Here, we present details on the construction of CKSAAP_UbSite, the overall performance assessment, and the intensive benchmark experiments against some existing predictors. In particular, why CKSAAP is suitable for the prediction of ubiquitination sites is also discussed.

## Methods

### Datasets

To construct CKSAAP_UbSite, 203 ubiquitylated substrates, which were previously compiled by Radivojac et al [2], were downloaded from http://www.ubpred.org/sgd_predictions.txt.gz. These 203 proteins contained 272 experimentally validated ubiquitination sites, which are regarded as positive samples. Generally, all the remaining K residues that were not reported as ubiquitination sites in these proteins can be regarded as negative samples (i.e. non-ubiquitination sites). It should be clearly pointed out that these remaining residues may contain ubiquitination sites that are not experimentally identified yet. By employing the similar strategy as the work of Radivojac et al [2], we extracted 4642 negative samples from the 124 mitochondrial matrix proteins. Since there is no chance for the mitochondrial matrix proteins accessible for the Ub-proteasome system [2], the reliability of the 4642 negative samples can be guaranteed. Thus, the 272 positive samples together with the 4642 negative samples were compiled into an initial dataset. As already mentioned in the Introduction section, each sample is represented by a sequence fragment with a window size of 2*n*+1. According to our preliminary computational experiments, the window size was optimally set as 27 in this study. In order to avoid the overestimation of performance caused by the sequence redundancy, we took the threshold of 40% sequence identity to filter the initial dataset. Briefly, the filtering ensured that any fragment pair in all the remaining positive and negative samples shared a sequence identity less than 40%. Finally, we obtained a filtered ubiquitination site dataset containing 263 positive and 4345 negative samples (i.e. Radivojac_dataset), which was used to train and test CKSAAP_UbSite (see Supporting Information Text S1).

### Encoding schemes and feature selection

#### The CKSAAP encoding scheme

In this study, an ubiquitination or non-ubiquitination site is represented by a sequence fragment of 27 amino acids. Thus, the CKSAAP encoding means the composition of *k*-spaced residue pairs in the fragment. Taking *k* = 0 as an example, there are 400 0-spaced residue pairs (i.e., AA, AC, AD,…, YY). Then, a feature vector can be defined as
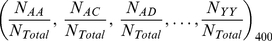
(1)


The value of each feature denotes the composition of the corresponding residue pair in the fragment. For instance, if the residue pair AA appears *m* times in the fragment, the composition of the residue pair AA is equal to *m* divided by the total number of *0*-spaced residue pairs (*N_Total_*) in the fragment. For *k* = 0, 1, 2, 3, 4 and 5, the value of *N_Total_* is 26, 25, 24, 23 and 22, respectively. In case a very few ubiquitination or non-ubiquitination sites are located in the N- or C-terminal of protein sequences, the corresponding values of *N_Total_* should be adjusted accordingly. Considering that the CKSAAP encoding was performed over *k* = 0, 1, 2, 3, 4 and 5 in this study, the total dimension of the CKSAAP-based feature vector is 2400.

#### The binary encoding scheme

To benchmark against the CKSAAP encoding scheme, the binary encoding scheme was also carried out. For the sites located in N- or C-terminal, the number of residues may be less than 27. To ensure the binary encoding with a unified dimension (i.e. each site should be represented by a sequence fragment of 27 residues), we assigned a non-existing amino acid O to fill in the corresponding positions. Thus, 21 different amino acids are considered in the binary encoding, which are ordered as ACDEFGHIKLMNPQRSTVWYO. Briefly, each amino acid is represented by a 21-dimensional binary vector, e.g.

A (100000000000000000000), C(010000000000000000000), … , O(010000000000000000001), etc. Because the central position is always K, it is not necessary to be taken into account. Therefore, the total dimension of the binary encoding scheme is 21×26 = 546.

#### Feature selection

Since the proposed CKSAAP encoding contains a large number of features, two well-established dimensionality reduction methods, Chi-Squared (CHI) [25] and Information Gain (IG) [22], [24], [25], were employed to rank the corresponding features in CKSAAP. Please refer to the literature[25] for more details about the CHI and IG-based feature selections. To avoid the potential over-fitting problem, it is worth mentioning that the feature selection procedures were stringently conducted. In particular, the testing samples should always be excluded from the feature selection procedures.

### SVM learning

As a machine-learning method of binary classification, SVM aims to find a rule that best maps each member of a training set to the correct classification [29], which has been used for diverse prediction/classification tasks related to protein bioinformatics [30]–[33]. Using the CKSAAP encoding as input, the SVM was trained to distinguish ubiquitination and non-ubiquitination sites in this study. The implemented SVM algorithm was SVM-light (http://svmlight.joachims.org/) and the applied kernel function was the radial basis function (RBF). In order to maximize the performance of the SVM algorithm, two parameters (i.e. the regularization parameter *C* and the width parameter *γ*) in the RBF kernel were preliminarily optimized through a grid search strategy. First, the range of *C* and γ was empirically set to be [0.5, 8.0] and [0.5, 16], respectively. Then, a step of 0.5 was assigned for *C* and γ, which resulted in a total number of 16×32 = 512 grids. Finally, all the 512 grids were evaluated to determine the optimal SVM parameters.

### Performance assessment of CKSAAP_UbSite

In this study, four measurements, i.e. Accuracy (*Ac*), Sensitivity (*Sn*), Specificity (*Sp*), and Matthew correlation coefficient (*MCC*) were used to evaluate the prediction performance. They are defined as:
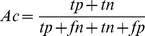
(2)

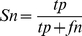
(3)

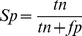
(4)

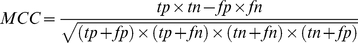
(5)where *tp*, *fp*, *fn* and *tn* represents the true positives, false positives, false negatives and true negatives, respectively. To provide a comprehensive understanding of the performance, we also used a Receiver Operating Characteristic (ROC) curve [34], [35], which plots the true positive rate (i.e. *Sn*) as a function of the false positive rate (i.e. 1-*Sp*) for all possible thresholds. Furthermore, the overall performance of CKSAAP_UbSite can also be quantified by the corresponding area under the ROC curve (AUC). Generally, the closer the AUC value is to 1, the better the performance is.

## Results and Discussion

### Performance of CKSAAP_UbSite

**Figure 1:**
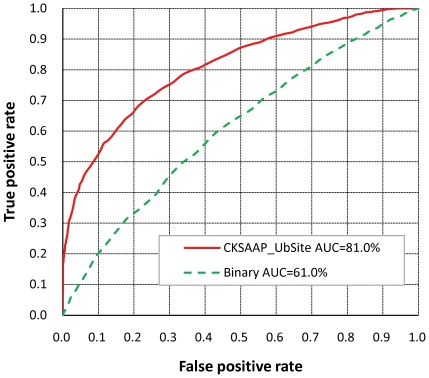
ROC curves of CKSAAP_UbSite and the binary encoding scheme based on balanced ubiquitination and non-ubiquitination sites. The performance of CKSAAP_UbSite and the binary encoding scheme was assessed through a 100-fold cross-validation strategy.

The proposed CKSAAP_UbSite predictor was trained and tested on a balanced dataset (i.e. 263 ubiquitination sites and 263 non-ubiquitination sites selected from Radivojac_dataset) through a 100-fold cross-validation. Since the number of available non-ubiquitination sites in Radivojac_dataset is much larger than that of ubiquitination sites, we repeated the above training/testing procedures 10 times by randomly changing the negative samples (see Supporting Information Text S1 for more details about the 10 different sets of negative samples). To have a stringent assessment of CKSAAP_UbSite, the same SVM parameters should be used in these 10 different sets. Therefore, we conducted the grid search on the 100-fold cross-validation through the 10 different sets. The parameters *C*  = 2.0 and γ = 8.0, which resulted in the best performance (i.e. the average *Ac* over all the cross-validation is the highest), were considered as the optimal SVM parameters of CKSAAP_UbSite. The average performance of CKSAAP_UbSite is summarized in Table 1. The detailed performance measurements for these 10 benchmark experiments are listed in Supporting Information Text S2. In general, the performance of CKSAAP_UbSite is reasonably good. The average *Ac* of CKSAAP_UbSite reached 73.40% (*Sn* = 69.85%, *Sp* = 76.96%, *MCC* = 0.4694) (Table 1). Furthermore, the ROC curve of CKSAAP_UbSite was plotted in Figure 1 and the corresponding value of AUC was 81.0%. At a less than 10% false positive rate control, CKSAAP_UbSite can correctly identify about 52.5% ubiquitination sites.

**Table 1:** Comparison of CKSAAP_UbSite with the binary encoding and UbPred.

Method	*Sn*(%)	*Sp*(%)	*Ac*(%)	*MCC*
CKSAAP_UbSite^a^	69.85±1.67	76.96±2.52	73.40±1.71	0.4694±0.0347
The binary encoding^a^	56.23±2.21	60.04±3.56	58.14±2.30	0.1630±0.0486
UbPred^b^	_ _	_ _	72.00	_ _

a The corresponding measurement was represented as the average value ± standard deviation.

b The corresponding value was cited from Radivojac et al (2010) [2]. ‘_ _’ means the corresponding value is not available.

Because of the high dimension of the CKSAAP encoding, two feature selection methods were conducted to find the most relevant features and to reduce the dimensionality of the encoding. It was observed that the improvements after both feature selections are negligible (data not shown), which could be ascribed to the following two aspects. First, SVM has a good tolerance to high dimensional data (i.e. SVM is not sensitive to the so called “the curse of dimensionality”). Second, the number of positive samples is too small and the selected features based merely on the training dataset could not reflect the overall characteristic around the ubiquitination sites.

To facilitate the community's research, a web server of CKSAAP_UbSite was constructed and is freely available at http://protein.cau.edu.cn/cksaap_ubsite/, which can be further used for proteome-wide ubiquitination site identification. To provide a more stable prediction result, 10 SVM predictors corresponding to 10 different sets of negative samples were jointly utilized. It should be emphasized that the optimal SVM parameters (i.e. *C*  = 2.0 and γ = 8.0) was used to construct these 10 SVM predictors. For a query site, the final prediction score is averaged over these 10 SVM outputs. In general, the predicted ubiquitination sites at a low false positive rate are more informative for practical applications. To quantitatively understand the reliability of the prediction, we provided the threshold values for two different confidence levels, which correspond to the false positive rates of 2% and 10%, respectively. It should be pointed out that the above two threshold values were based on a balanced dataset. In fact, the ubiquitination and non-ubiquitination sites in proteins are highly unbalanced. For example, the ratio of ubiquitination to non-ubiquitination sites in Radivojac_dataset is approximately 1:17. For practical applications, more stringent threshold values should be suggested to guarantee the prediction results at a low false positive rate control.

### The significant features

**Figure 2:**
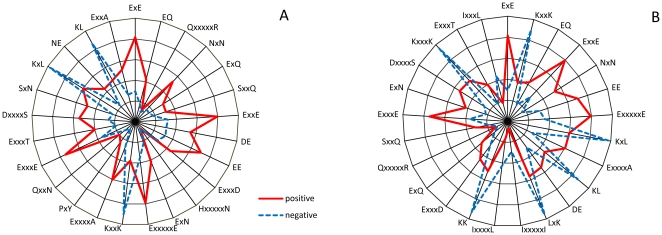
The composition of the top-25 residue pairs resulting from two feature selection methods. The composition of each residue pair is represented by a radial vector whose length is proportional to the composition concerned.

**Figure 3:**
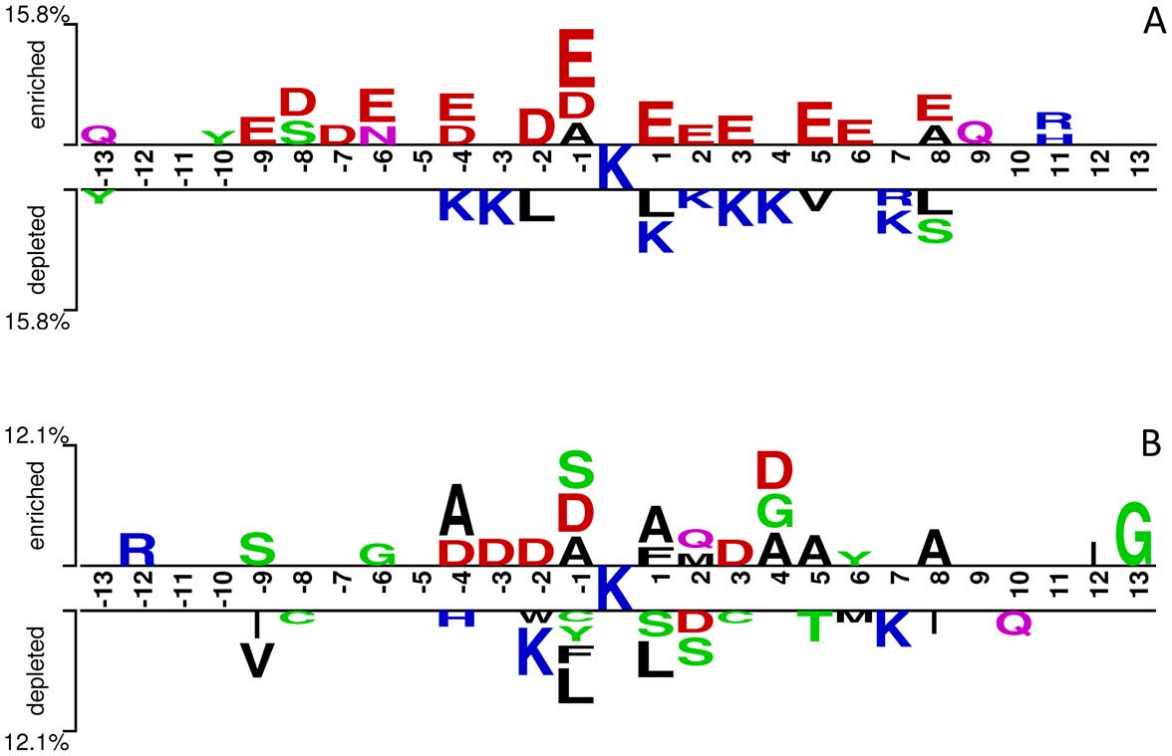
Two Two-Sample-Logos of the position-specific residue composition surrounding the ubiquitination sites and non-ubiquitination sites, which were inferred from Radivojac_dataset (A) and Cai_dataset_1 (B), respectively. These two logos were prepared using the web server http://www.twosamplelogo.org/ and only residues significantly enriched and depleted surrounding ubiquitination sites (*t*-test, *P*<0.05) are shown.

Although the two feature selection methods did not result in performance improvement, they allowed us to pick up most important features (i.e. *k*-spaced residue pairs). According to the output of the CHI- and IG- based feature selection methods, the corresponding top-25 residue pairs are listed in Table 2. The composition of the top-25 residue pairs were also presented in two radar diagrams (Figure 2). As can be seen from Figure 2, the composition of these top-25 features, either inferred from CHI- or IG-based feature selection, are remarkably different in ubiquitination and non-ubiquitination sites. Interestingly, there are 19 residue pairs appearing in the two top-25 feature subsets, implying a good consistency between these two feature selection methods. The importance of these 19 residue pairs is also clearly and intuitively characterized in Figure 3A. For instance, the feature ‘ExE’, which represents the ‘EE’ residue pair spaced by any amino acid (i.e. 1-spaced residue pair), is significantly enriched in position pairs (−6/−4, −1/+1, +1/+3, +3/+5 and +6/+8) surrounding the ubiquitination sites. As another example, the important of ‘KL’ is also represented by its depleted occurrence in some position pairs (−3/−2 and +7/+8) around the ubiquitination sites. In addition to providing some explanations about the powerfulness of the CKSAAP encoding, the important residue pairs listed in Table 2 may also offer some new clues for the sequence patterns around the ubiquitination sites, which indeed deserve for further experimental validation.

**Table 2:** The top 25 features ranked by CHI- and IG- based feature selection methods.

Top 25 features	CHI	IG
1	**E**x**E** a ^,^ b	**E**x**E**
2	**E**Q	**K**xx**K**
3	**Q**xxxxx**R**	**EQ**
4	**N**x**N**	**E**xx**E**
5	**E**x**Q**	**N**x**N**
6	**S**xx**Q**	**EE**
7	**E**xx**E**	**E**xxxxx**E**
8	**DE**	**K**x**L**
9	**EE**	**E**xxxx**A**
10	**E**xxx**D**	**KL**
11	HxxxxxN	**DE**
12	**E**x**N**	LxK
13	**E**xxxxx**E**	IxxxxxI
14	**K**xx**K**	IxxxxxL
15	**E**xxxx**A**	KK
16	PxY	**E**xxx**D**
17	QxxN	**E**x**Q**
18	**E**xxx**E**	**Q**xxxxx**R**
19	**E**xxx**T**	**S**xx**Q**
20	**D**xxxx**S**	**E**xxx**E**
21	SxN	**E**x**N**
22	**K**x**L**	**D**xxxx**S**
23	NE	KxxxK
24	**KL**	**E**xxx**T**
25	ExxA	IxxxL

a The feature ‘ExE’ represents a 1-spaced residue pair of ‘EE’, where x stands for any amino acid. The same representation was applied to other *k*-spaced residue pairs.

b The *k*-spaced amino acid pairs in bold type mean they are consistently ranked as the top-25 features by both feature selection methods.

### Comparison with the binary encoding scheme

When compared with the binary encoding scheme by using the same dataset (i.e. Radivojac_dataset), the proposed CKSAAP encoding revealed about 15% higher *Ac* and a nearly 0.30 increment of *MCC* (Table 1). The better performance of the proposed CKSAAP encoding was further illustrated by the ROC analysis (Figure 1), in which CKSAAP_UbSite outperformed the binary encoding by showing about 0.20 higher AUC value. All the above results clearly showed that the CKSAAP encoding has a significant advantage over the binary encoding in predicting ubiquitination sites.

In general, the binary encoding characterizes the position-specific feature of a sequence fragment. In other words, the binary encoding would perform well in case that the fragments surrounding the ubiquitination sites have some position-specific conservation patterns. On the contrary, the CKSAAP encoding pays attention on the collocation of amino acid pairs at different positions surrounding ubiquitnation sites, which can also reflect the composition of short linear motifs[36] to some extent. Often residing in disordered regions, these short linear motifs contain three to eight residues, in which two or three key residues are conserved [36]. The short linear motifs have been widely reported to be involved in many biological processes such as the communication of protein-protein interaction [36]. Compared with the binary encoding scheme, the better performance of CKSAAP_UbSite implied that short linear motifs maybe more important than position-specific patterns in recognizing ubiquitylated substrates. Since the binary encoding scheme or position-specific sequence features have been widely used in diverse PTM site prediction tasks [37]–[39], we might also expect a better performance of the CKSAAP encoding in the prediction of other PTM sites. In fact, we have experienced a more powerful performance of the CKSAAP encoding in mucin-type O-glycosylation site prediction [16], while its performance in predicting phosphorylation and sumoylation sites did not outperform the binary encoding scheme (data not shown). It is also worth mentioning that the CKSAAP encoding has been reported to predict the structural property of a sequence fragment [22]. Therefore, the performance of CKSAAP_UbSite may further imply that some structural constraints are required for ubiquitination sites.

### Comparison of CKSAAP_UbSite with three existing predictors

**Figure 4:**
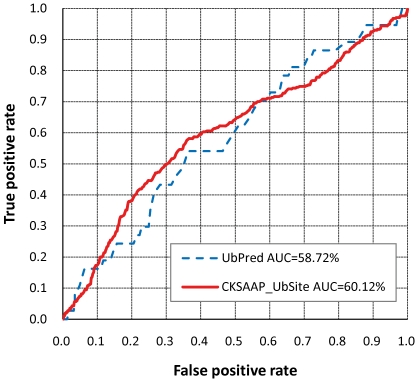
Comparison of CKSAAP_UbSite and UbPred based on an independent dataset of 21 proteins.

The proposed CKSAAP_UbSite was firstly benchmarked against UbPred. Since CKSAAP and UbPred are based on the same dataset and they adopted the same ratio of positive to negative samples (1∶1), which allowed a comparatively fair assessment between these two predictors. As shown in Table 1, the performance of CKSAAP_UbSite is reasonably better than UbPred by showing 1.4% higher prediction accuracy. To complement the comparison, we also conducted a benchmark experiment between CKSAAP_UbSite and UbPred on an independent test dataset. The test set was compiled through our literature reading, which covers 21 ubiquitylated proteins experimentally reported in the past two years. The test set contains 37 ubiquitination sites and 639 non-ubiquitination sites (Supporting Information Text S3). To conduct a comparison on this test set, these 21 proteins were processed via the web servers of CKSAAP_UbSite and UbPred, and the results were characterized by the ROC analysis. As shown in Figure 4, CKSAAP_UbSite generally outperforms UbPred by showing a nearly 0.014 higher AUC value, although CKSAAP_UbSite results in slightly lower true positive rates at low false positive rate controls. Surprisingly, both CKSAAP_UbSite and UbPred reveal dramatically lower performance on this test set when compared with the corresponding performance tested on Radivojac_dataset, implying that the sequence patterns around ubiquitination sites in Radivojac_dataset and the 21 proteins are remarkably different. Since these 21 proteins were mainly selected from the proteome of human, the current CKSAAP_UbSite and UbPred predictors, which were mainly inferred from yeast proteins, may be not fully suitable for the ubiquitination site identification of these 21 proteins.

We also compared CKSAAP_UbSite with a newly predictor developed by Cai et al (2011) [21]. Cai et al's method was trained and tested on a dataset of 364 ubiquitination sites and 1092 non-ubiquitination sites (i.e. Cai_dataset_1), which covers ubiquitylated substrates from diverse species. Approximately 50% and 35% ubiquitylated substrates in Cai_dataset_1 were collected from the proteomes of human and yeast, respectively. The ratio of ubiquitination sites to non-ubquitination sites in Cai et al's method was set to 1∶3 and the jackknife cross-validation was conducted. Furthermore, Cai et al's method was also tested in 12 independent proteins (i.e. Cai_dataset_2), which contain 14 ubiquitination sites and 267 non-ubiquitination sites. To have a fair comparison between CKSAAP_UbSite and Cai et al's method, we retrained CKSAAP_UbSite on Cai_dataset_1 and characterized the performance on the jackknife cross-validation. To save computational time, the default parameters (*C* = 1.9405 and γ = 1.0) of the RBF kernel in SVM training were employed in this benchmark experiment. Meanwhile, we also tested the performance on Cai_dataset_2. In general, CKSAAP_UbSite outperformed Cai et al's method considerably in both of the jackknife cross-validation and the test on Cai_dataset_2 (Table 3). Compared with the performance of CKSAAP_UbSite based on Radivojac_dataset, the performance tested on Cai_dataset_1 and Cai_dataset_2 is much poorer. To rule out the possibility that the decreased performance was caused by the different ratios of ubiquitination and non-ubiquitination sites, we also retrained and tested the performance of CKSAAP_UbSite with a 1∶3 ratio of ubiquitination sites to non-ubiquitination sites in Radivojac_dataset. Even with the same ratio of positive to negative samples as Cai_dataset_1, CKSAAP_UbSite performed much better in Radivojac_dataset than in Cai_dataset_1 (Table 3). Since Cai_dataset_1 was selected from different proteomes, Radivojac_dataset and Cai_dataset_1 have remarkably different sequence patterns around ubiquitination sites (Figure 3A and B). In line with the poor performance of UbPred and CKSAAP_UbSite in our manually-curated test set (Figure 4), the decreased performance in Cai_dataset_1 may imply that the sequence patterns around ubiquitination sites are not conserved across different organisms. Therefore, the development of organism-specific ubiquitination site predictor is necessary to obtain the maximal performance.

**Table 3:** Comparison of CKSAAP_UbSite with other predictors.

Method	Dataset	Ratio	*Sn*(%)	*Sp*(%)	*Ac*(%)	*MCC*
CKSAAP_UbSitea,b	Radivojac_dataset	1∶3	20.38	98.48	78.95	0.3374
CKSAAP_UbSitea,c	Cai_dataset_1	1∶3	6.74	99.53	76.33	0.1923
CKSAAP_UbSite^a^	Cai_dataset_2	1∶3	7.14	100.00	95.37	0.2610
Cai et alc,d	Cai_dataset_1	1∶3	_ _	_ _	_ _	0.1420
Cai et al^d^	Cai_dataset_2	1∶3	_ _	_ _	_ _	0.1390
UbPred^d^	Cai_dataset_2	1∶3	_ _	_ _	_ _	0.1350
Tung and Ho^d^	Cai_dataset_2	1∶3	_ _	_ _	_ _	0.1170

a To save computational time, the default parameters of the RBF kernel in SVM training were employed in this benchmark experiment.

b The result was based on the 100-fold cross-validation.

c The result was based on the jackknife cross-validation.

d The corresponding value was cited from Cai et al (2011) [21]. ‘_ _’ means the corresponding value is not available.

We also compared CKSAAP_UbSite with the predictor proposed by Tung and Ho (2008) indirectly. As reported by Radivojac et al (2010), UbPred outperformed Tung and Ho's method when tested on some newly identified ubiquitination sites. Moreover, Cai et al (2011) also benchmarked their method against Tung and Ho's method on the independent test set (i.e. Cai_dataset_2) and showed higher *MCC* (Table 3). Since CKSAAP_UbSite has been benchmarked to have better performance than UbPred and Cai et al's method, it is reasonable to believe that CKSAAP_UbSite should also be more powerful than Tung and Ho's method. All the three existing methods are statistical- or machine learning-based predictors and they employed hybrid sequence features. Compared with these three predictors, it is worth mentioning that the formula of the CKSAAP encoding is much more concise, although the dimension of the CKSAAP is still higher than the other feature vectors employed in the three peer predictors. More importantly, the reasonably good performance of CKSAAP_UbSite reflected that the CKSAAP encoding can effectively capture the information of enriched/depleted residue pairs around ubiquitination sites.

### Conclusion

In order to detect ubiquitination sites in query proteins, we developed a SVM-based predictor termed as CKSAAP_UbSite, which has been benchmarked to have better performance than some other existing predictors. With the ability of reflecting the sequence patterns surrounding the ubiquitination sites, the CKSAAP encoding has been proved to be particularly suitable for the prediction of ubiquitination sites. To facilitate the biological community, a web-server of CKSAAP_UbSite was constructed, which can be freely accessible at http://protein.cau.edu.cn/cksaap_ubsite/. Considering that the sequence patterns around ubiquitination sites in different organisms are not conserved, the real-world applications of the current predictor should be limited to the proteome of yeast. With the increment of experimentally verified ubiquitination sites in the near future, we forecast that more attention will be paid on the development of organism-specific predictors in order to maximize the prediction performance of ubiquitination sites.

## Supporting Information

Text S1 This file contains 263 ubiquitination and 4345 non-ubiquitination sites (i.e. Radivojac_dataset). Additionally, the 10 subsets of non-ubiquitination sites selected from the 4345 negative samples were also listed.
(TXT)

Text S2 This file shows the performance measurements of CKSAAP_UbSite for 10 different negative samples.
(DOC)

Text S3 This file contains an independent test set covering 37 ubiquitination sites and 639 non-ubiquitination sites.
(TXT)
