# Supplementary material for: Prediction of Ubiquitination Sites by Using the Composition of k-Spaced Amino Acid Pairs
Source: PLoS One. 2011 Jul 29;6(7):e22930. doi: 10.1371/journal.pone.0022930 (PMC3146527; doi:10.1371/journal.pone.0022930)
Supplement: Text S2 — This file shows the performance measurements of CKSAAP_UbSite for 10 different negative samples. (DOC) [file pone.0022930.s002.doc]

|  | **Table S1.** Prediction accuracy of CKSAAP_UbSite for 10 different negative samples and the corresponding parameters | | | | | | |  |
| --- | --- | --- | --- | --- | --- | --- | --- | --- |
| **Sample** | | **Sensitivity (%)** | **Specificity (%)** | **Accuracy (%)** | **MCC** | ***C*** | ***γ*** | |
| **1** | | 68.44 | 76.05 | 72.24 | 0.4462 | 2.0 | 8.0 | |
| **2** | | 67.30 | 74.90 | 71.10 | 0.4233 | 2.0 | 8.0 | |
| **3** | | 70.34 | 80.99 | 75.67 | 0.5162 | 2.0 | 8.0 | |
| **4** | | 67.68 | 76.43 | 72.05 | 0.4428 | 2.0 | 8.0 | |
| **5** | | 70.72 | 73.38 | 72.05 | 0.4412 | 2.0 | 8.0 | |
| **6** | | 71.86 | 76.43 | 74.14 | 0.4834 | 2.0 | 8.0 | |
| **7** | | 71.10 | 77.19 | 74.14 | 0.4838 | 2.0 | 8.0 | |
| **8** | | 68.82 | 78.33 | 73.57 | 0.4736 | 2.0 | 8.0 | |
| **9** | | 71.86 | 80.99 | 76.43 | 0.5307 | 2.0 | 8.0 | |
| **10** | | 70.34 | 74.90 | 72.62 | 0.4529 | 2.0 | 8.0 | |
| **Average** | | 69.85 ± 1.67 | 76.96 ± 2.52 | 73.40 ± 1.71 | 0.4694 ± 0.0346 | - - | - - | |
